# Microdosimetric characterization of proton relative biological effectiveness in pancreatic ductal adenocarcinoma cell lines

**DOI:** 10.3389/fonc.2026.1934487

**Published:** 2026-09-16

**Authors:** Tianyu Wang, Yuting Li, Sabrina N. Campelo, Lawrence Bronk, Paige A. Taylor, Tara N. Fujimoto, Dadi Jiang, Albert C. Koong, Fada Guan

**Affiliations:** 1 Department of Radiation Oncology, The University of Texas MD Anderson Cancer Center, Houston, TX, United States; 2 Cancer Biology Program, The University of Texas MD Anderson Cancer Center UTHealth Houston Graduate School of Biomedical Sciences, Houston, TX, United States; 3 Department of Radiation Physics, The University of Texas MD Anderson Cancer Center, Houston, TX, United States

**Keywords:** crystal violet assay, microdosimetry, pancreatic ductal adenocarcinoma, proton therapy, relative biological effectiveness

## Abstract

**Purpose:**

Pancreatic ductal adenocarcinoma (PDAC) is among the most radioresistant malignancies. Proton therapy assumes a uniform relative biological effectiveness (RBE) of 1.1 regardless of radiation quality. We characterized proton RBE as a function of dose-mean lineal energy (*ȳ_D_*) in PDAC cell lines.

**Methods:**

Proton RBE was assessed in Panc-1 and COLO 357 FG cells using a high-throughput 96-well irradiation platform with an 81.4 MeV spot-scanning proton beam. Cells were irradiated at 12 discrete depths along the pristine Bragg curve, spanning *ȳ_D_* values of 1.9 to 20.0 keV/μm. Dose was varied by adjusting spot-scanning repaintings. Parallel 6 MV photon irradiations (*ȳ_D_* = 0.8 keV/μm) served as reference. Cell viability was assessed by crystal violet assay and fitted with the linear-quadratic (LQ) model to extract RBE at 10% viability.

**Results:**

Both cell lines demonstrated RBE near 1.0 at entrance energies (*ȳ_D_* < 10 keV/μm). Beyond the Bragg peak, RBE rose sharply: for Panc-1, RBE reached 3.0 ± 0.3 at *ȳ_D_* = 18.7 keV/μm and 4.6 ± 0.4 at *ȳ_D_* = 20.0 keV/μm; for COLO 357 FG, RBE reached 3.6 ± 0.2 and 6.1 ± 0.5 at the same positions. For Panc-1, LQ fits were good across all conditions. In contrast, COLO 357 FG exhibited a linear dose-response (β≈0) for protons despite a clear LQ response to photons, suggesting proton-induced damage complexity exceeds the repair capacity of this cell line.

**Conclusions:**

The clinical RBE = 1.1 assumption substantially underestimates proton effectiveness in the distal Bragg peak region for PDAC. This microdosimetric RBE dataset provides a quantitative basis for improving proton treatment planning in radioresistant PDAC.

## Introduction

Pancreatic ductal adenocarcinoma (PDAC) is a highly lethal malignancy, with a 5-year survival rate of only 8% and a prognosis that has improved only modestly over the past two decades (1). Radiation therapy plays an important role in locally advanced PDAC, but the inherent radioresistance of PDAC limits treatment efficacy even with concurrent chemotherapy (2). Proton therapy offers a dosimetric advantage through the Bragg peak, which concentrates dose deposition at depth with no exit dose, making it a promising option for PDAC, where proximity to critical abdominal structures limits conventional photon therapy. However, the biological effectiveness of proton therapy in PDAC remains poorly characterized.

Proton therapy currently assigns a uniform relative biological effectiveness (RBE) value of 1.1 relative to photons, regardless of proton energy or tissue type (3). This assumption is increasingly challenged by experimental evidence demonstrating that RBE varies substantially along the proton Bragg curve, rising sharply in the distal fall-off region (4, 5). Although the physical quantity of linear energy transfer (LET) has been widely used as a surrogate for proton biological effectiveness, its limitations have been demonstrated experimentally: the same dose-averaged LET can yield distinctly different RBE values depending on the spectral shape of the energy deposition distribution (6). Microdosimetry is recommended as a more rigorous framework for characterizing radiation quality in particle therapy, as microdosimetric quantities capture the stochastic distribution of energy deposition at the cellular level (7). Microdosimetry-based RBE models, including the modified microdosimetric kinetic model (MKM) (8), its extension SMK (9), and the recently validated generalized stochastic microdosimetric model (GSM^2^) (10), have demonstrated improved predictive accuracy for biological effectiveness across proton, helium, and carbon ion beams compared with LET-based approaches.

Despite growing evidence of spatial variation in proton RBE, no microdosimetric RBE characterization exists for PDAC cell lines. Given the inherent radioresistance of PDAC and the growing clinical interest in particle therapy for this disease, understanding how proton radiation quality influences biological effectiveness in PDAC is essential. We therefore performed the systematic spatial mapping of proton RBE along the pristine Bragg curve with microdosimetric characterization in two PDAC cell lines using a high-throughput irradiation platform.

## Methods

### Cell lines and culture conditions

The human PDAC cell line Panc-1 was purchased from Sigma-Aldrich (St. Louis, MO, USA), and COLO 357 FG cell line was a gift from Dr. Rosa F. Hwang at MD Anderson Cancer Center (11). All cell lines were free of mycoplasma at the time of the assay. Neither cell line is present in the International Cell Line Authentication Committee (ICLAC) database of commonly misidentified cell lines (version 14). Cell lines were cultured in Dulbecco’s modified Eagle’s medium (DMEM) with 10% (volume/volume; v/v) heat-inactivated bovine serum HI-FBS (Gibco) and 1% (v/v) penicillin/streptomycin (Gibco) at 37°C and 5% CO_2_. Cells were seeded into 96-well plates at a density of 1,500 cells per well and allowed to adhere overnight 24 hours before irradiation.

### High-throughput spatial mapping irradiation platform

Cells were irradiated with a high-throughput, high-definition 96-well plate irradiation platform designed for spatial mapping of biological effectiveness along the proton Bragg curve (4, 12). The platform is constructed from a multi-step Lucite block, with each step providing an incremental water-equivalent thickness to position cells at 12 discrete depths along the Bragg curve of an 81.4 MeV spot-scanning proton beam simultaneously. The platform has sub-millimeter spatial resolution between adjacent step positions to finely sample the narrow Bragg peak region. This high spatial resolution, combined with the 96-well format (8 rows × 12 columns), enables efficient and comprehensive characterization of RBE across the full range of radiation qualities from beam entrance through the distal edge in a single experiment. Dose was varied by adjusting the number of repaintings in the scan pattern. The dose per repainting at each of the 12 positions along the Bragg curve was: 0.027, 0.059, 0.091, 0.109, 0.116, 0.127, 0.134, 0.131, 0.122, 0.106, 0.073, and 0.050 Gy for columns 1 through 12, respectively. As the reference radiation, 6 MV photon irradiations were performed in parallel on a clinical linear accelerator. The microdosimetric quantity dose-mean lineal energy (*ȳ_D_*) at the cellular level was calculated using experimentally validated Geant4 Monte Carlo simulations (4, 6, 12), modeling a 2-μm diameter water-filled sphere to represent the cell nucleus, yielding *ȳ_D_* = 0.8 keV/μm for 6 MV photons and *ȳ_D_* values ranging from 1.9 to 20.0 keV/μm across the 12 proton positions. The relative error of *ȳ_D_* is < 0.15%, and the relative error of dose is < 0.05% from Monte Carlo simulations. Our calculated *ȳ_D_* values are in good agreement with experimentally measured microdosimetric data for proton beams of comparable energy (13). Monte Carlo-based microdosimetric calculations for proton beams have also been reported in the literature (14).

### Crystal violet cell viability assay

Crystal violet staining was performed at approximately 120 hours post-irradiation, the time point at which control (unirradiated) wells reached confluence. Both irradiated and unirradiated 96-well plates were fixed with ice-cold methanol and stained with a 0.25% (weight/volume; w/v) crystal violet solution for 30 minutes. The plates were then gently rinsed with tap water until no background staining remained visible. The plates were then photographed, the bound crystal violet was solubilized in 33% (v/v) acetic acid, and absorbance was quantified at 590 nm with an Epoch microplate reader.

### Linear-quadratic model fitting and RBE calculation of cell viability

Dose-response data were fitted with the linear-quadratic (LQ) model: S = exp(−αD − βD^2^), where S represents the relative cell viability, D is dose, and α and β are fitting parameters. Independent α and β parameters were fitted (weighted by 1/Y^2^) for each *ȳ_D_* position for protons and for 6 MV photons using GraphPad Prism (v10.6.1). Proton RBE was calculated at 10% viability with 6 MV photon data as the reference. Uncertainties in RBE were propagated from the 95% confidence intervals of α and β. All results were reported as mean ± SEM.

## Results

The biological impact of proton radiation quality is visually demonstrated in **Figure 1A**, where column C4 in the entrance-to-proximal region and column C10 in the Bragg peak-to-distal edge region received nearly equivalent doses (~5.4 Gy) but had markedly different *ȳ_D_* values (6.2 vs. 16.3 keV/μm) at 50 repaintings. Despite the similar physical dose, wells in C10 showed substantially reduced cell density relative to those in C4, providing direct visual evidence that proton biological effectiveness increases with radiation quality along the Bragg curve. The corresponding microdosimetric lineal energy dose spectra across all 12 depth positions [water equivalent depths: 0.14, 4.24, 4.70, 4.79, 4.81, 4.84, 4.92 (Bragg peak), 4.96, 4.98, 5.00, 5.02, and 5.04 cm] are shown in **Figure 1B**, demonstrating the progressive rightward shift of the spectral peak with increasing depth as protons slow down toward and beyond the Bragg peak.

**Figure 1:**
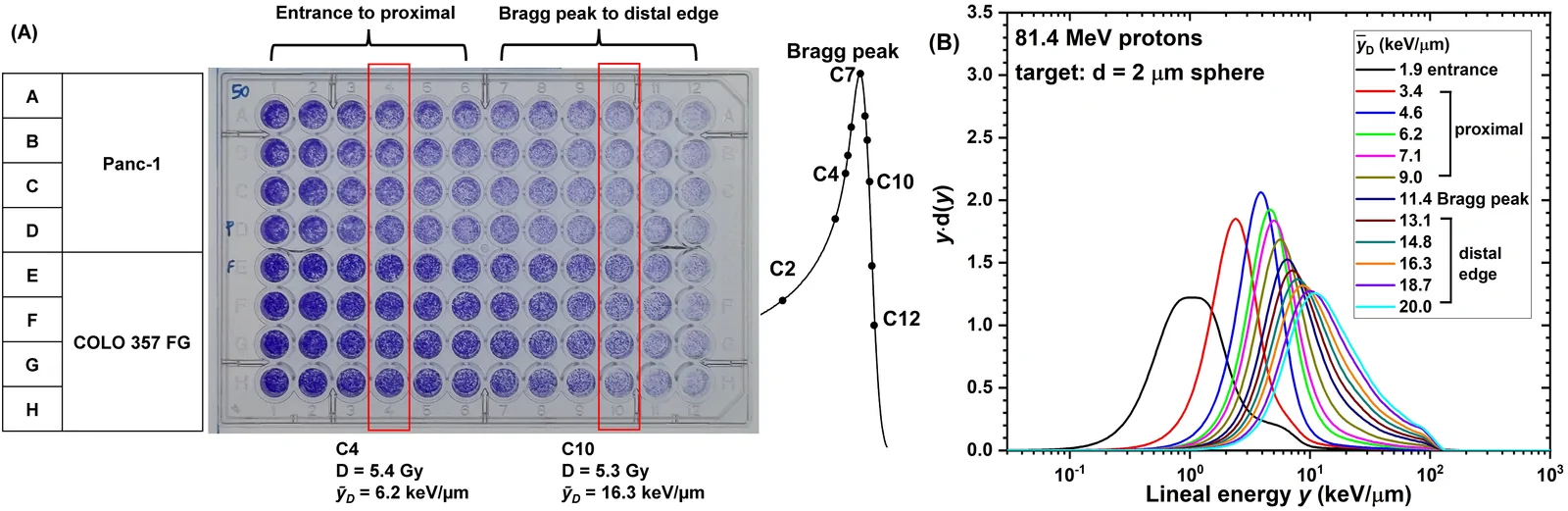
**(A)** Representative crystal violet-stained 96-well plate (50 proton repaintings) showing Panc-1 [rows **(A–D)**] and COLO 357 FG [rows **(E–H)**]. Red boxes highlight columns C4 [**(D)** = 5.4 Gy, *ȳ_D_* = 6.2 keV/μm, entrance-to-proximal region] and C10 (D = 5.3 Gy, *ȳ_D_* = 16.3 keV/μm, Bragg peak-to-distal edge region), demonstrating greater cell killing at higher lineal energy despite nearly equivalent doses. On the right: schematic of the 81.4 MeV proton pristine Bragg curve with column positions indicated. **(B)** Microdosimetric lineal energy dose spectra for 81.4 MeV protons at 12 depth positions along the pristine Bragg curve, calculated using Geant4 Monte Carlo simulations for a 2-μm diameter water-filled sphere mimicking the cell nucleus at each position of the 96-well plate irradiation platform.

### Relative viability of PDAC cells

Both Panc-1 and COLO 357 FG demonstrated clear LQ dose-response relationships to 6 MV photon irradiation with similar radiosensitivity (**Figure 2**). Proton dose-response curves were measured at 12 discrete depth positions spanning the entrance region through the distal edge of the Bragg curve (**Figures 3A, B**). Data from column C1 (*ȳ_D_* = 1.9 keV/μm) for Panc-1 and columns C1 and C2 (*ȳ_D_* = 1.9 and 3.4 keV/μm) for COLO 357 FG cells were excluded from the analysis owing to large fluctuations in viability inconsistent with a monotonic dose-response relationship. For Panc-1, the LQ model provided good fits across all remaining positions (**Figure 3A**). Strikingly, COLO 357 FG exhibited a linear dose-response (β≈0) for most proton conditions despite retaining a clear LQ response to 6 MV photons (**Figure 3B**), suggesting that proton-induced damage complexity exceeds the sublethal damage repair capacity of this cell line.

**Figure 2:**
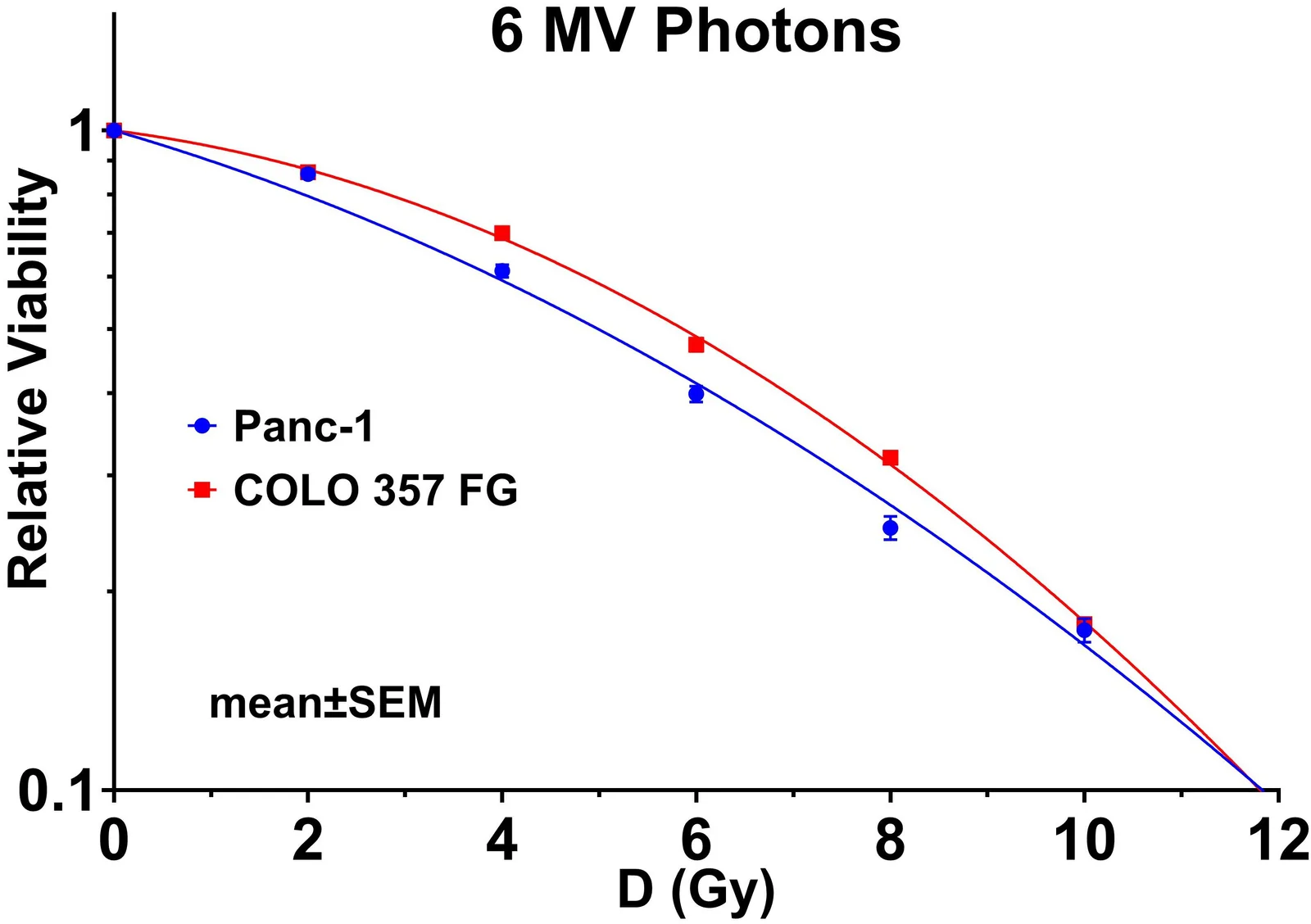
Relative viability of Panc-1 and COLO 357 FG cells under 6 MV photon irradiation. Data are mean ± SEM. n = 22 technical replicates (individual wells) for each data point. Adjusted R^2^ = 0.9370 and 0.9774 for Panc-1 and COLO 357 FG cells, respectively.

**Figure 3:**
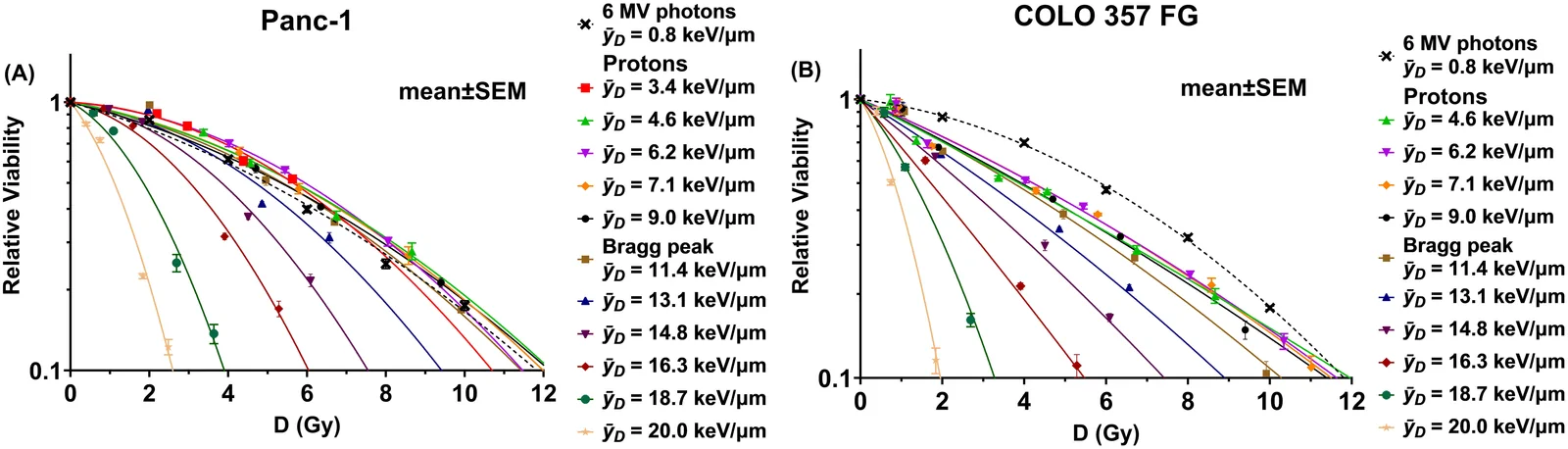
Relative viability of Panc-1 cells **(A)** and COLO 357 FG cells **(B)** as a function of proton dose at different positions along the 81.4 MeV Bragg curve, with 6 MV photons as reference (dashed line). Lines represent LQ model fits. Note: data from *ȳ_D_* = 1.9 keV/μm were excluded for Panc-1 cells and data from *ȳ_D_* = 1.9 and 3.4 keV/μm were excluded for COLO 357 FG cells owing to large fluctuation in viability. Data are mean ± SEM. n = 4 technical replicates (individual wells) for each data point. Adjusted R^2^ ranged from 0.9447 to 0.9895 and from 0.9128 to 0.9798 for Panc-1 and COLO 357 FG cells, respectively.

### RBE as a function of microdosimetric beam quality

RBE at 10% viability remained near 1.0 for both cell lines from the beam entrance through the Bragg peak (*ȳ_D_* < 12 keV/μm) (**Figure 4**). Beyond the Bragg peak, RBE rose sharply with increasing *ȳ_D_* for both cell lines. For Panc-1, RBE reached 1.6 ± 0.1 at *ȳ_D_* = 14.8 keV/μm, 3.0 ± 0.3 at *ȳ_D_* = 18.7 keV/μm, and 4.6 ± 0.4 at *ȳ_D_* = 20.0 keV/μm. For COLO 357 FG, the increase was steeper: RBE reached 1.6 ± 0.1, 3.6 ± 0.2, and 6.1 ± 0.5 at *ȳ_D_* = 14.8, 18.7, and 20.0 keV/μm, respectively. The higher RBE values observed in COLO 357 FG relative to Panc-1 at equivalent *ȳ_D_* positions are consistent with its linear proton dose-response, where the absence of a quadratic repair component results in steeper effective killing per unit dose compared with Panc-1. RBE, LQ model fit parameters α and β values (mean ± SEM) for all positions are summarized in **Table 1**. While **Figure 4** and **Table 1** report RBE values with SEM, the derived 95% confidence intervals (RBE ± 1.96×SEM) overlap between the two cell lines at all *ȳ_D_* positions, indicating that the observed RBE differences do not reach statistical significance at any individual position; however, both cell lines show statistically significant deviation from the clinical RBE = 1.1 assumption from *ȳ_D_* = 13.1 keV/μm onward for COLO 357 FG and from *ȳ_D_* = 14.8 keV/μm onward for Panc-1, where the lower bound of the 95% CI exceeds 1.1.

**Figure 4:**
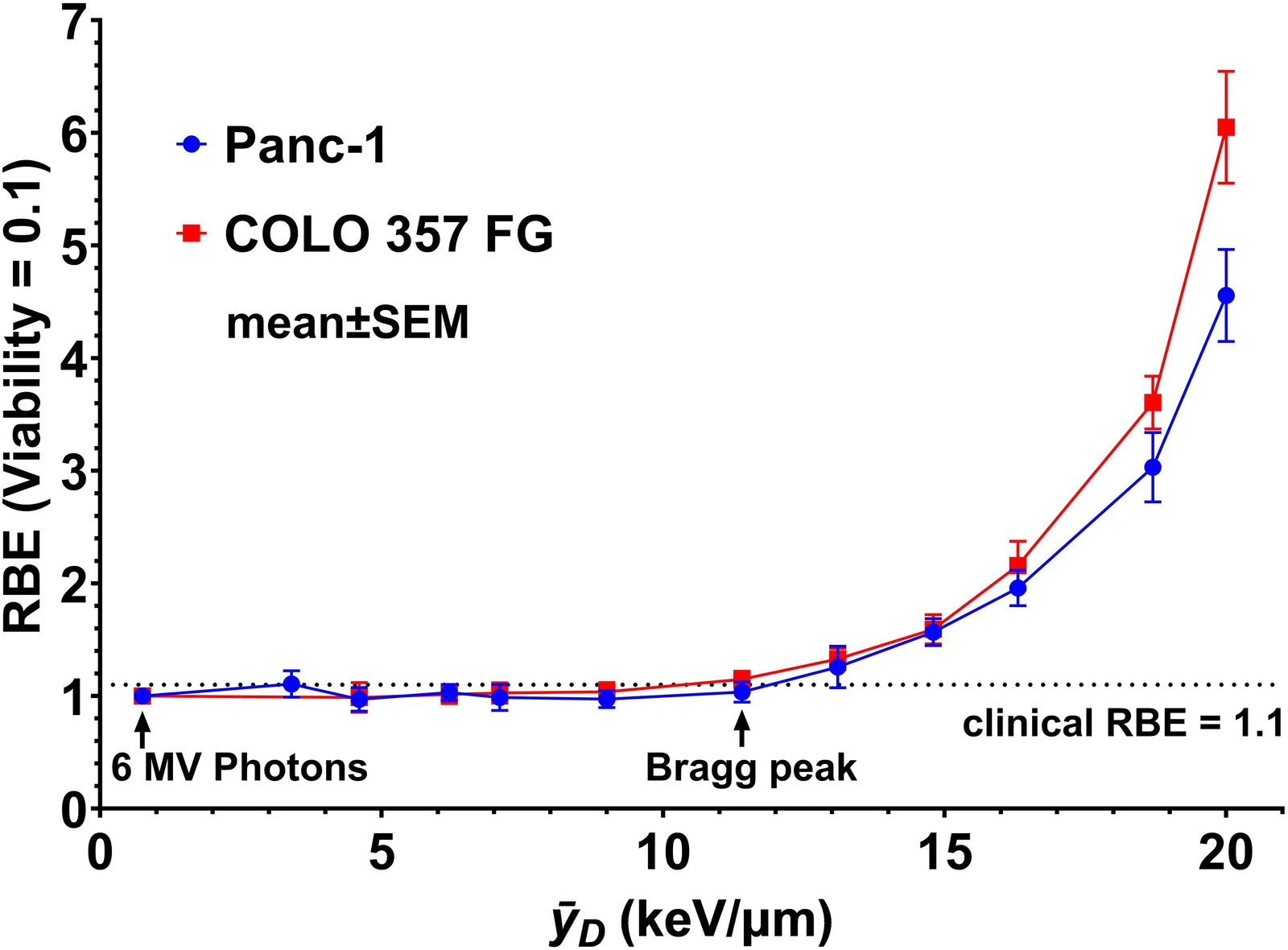
RBE at viability=0.1 as a function of *ȳ_D_* for Panc-1 and COLO 357 FG cells. The dotted line indicates the clinical RBE = 1.1 assumption. RBE remains near 1.0 from entrance to Bragg peak and increases sharply beyond Bragg peak, reaching 4.6 ± 0.4 and 6.1 ± 0.5 for Panc-1 and COLO 357 FG cells, respectively, at *ȳ_D_* = 20.0 keV/μm.

**Table 1:** Linear-quadratic model parameters (α, β) and RBE at 10% viability for Panc-1 and COLO 357 FG pancreatic ductal adenocarcinoma cell lines as a function of dose-mean lineal energy (*ȳ_D_*). Values are mean ± SEM.

Position	*ȳ_D_* (keV/µm)	Panc-1			COLO 357 FG		
		RBE	*α* (Gy^-1^)	*β* (Gy^-2^)	RBE	*α* (Gy^-1^)	*β* (Gy^-2^)
1	1.9	NA	NA	NA	NA	NA	NA
2	3.4	1.11 ± 0.12	0.019 ± 0.016	0.018 ± 0.003	NA	NA	NA
3	4.6	0.97 ± 0.10	0.063 ± 0.016	0.010 ± 0.002	0.99 ± 0.13	0.172 ± 0.015	0.002 ± 0.002
4	6.2	1.03 ± 0.07	0.026 ± 0.009	0.015 ± 0.001	1.02 ± 0.08	0.145 ± 0.012	0.005 ± 0.001
5	7.1	0.99 ± 0.12	0.059 ± 0.018	0.011 ± 0.002	1.03 ± 0.09	0.143 ± 0.014	0.005 ± 0.001
6	9.0	0.97 ± 0.08	0.083 ± 0.010	0.009 ± 0.001	1.04 ± 0.08	0.163 ± 0.012	0.003 ± 0.001
7 (Bragg peak)	11.4	1.03 ± 0.09	0.064 ± 0.015	0.012 ± 0.002	1.15 ± 0.07	0.163 ± 0.012	0.006 ± 0.001
8	13.1	1.26 ± 0.19	0.052 ± 0.032	0.021 ± 0.005	1.33 ± 0.09	0.205 ± 0.016	0.006 ± 0.002
9	14.8	1.57 ± 0.12	0.052 ± 0.019	0.034 ± 0.003	1.59 ± 0.13	0.263 ± 0.022	0.006 ± 0.002
10	16.3	1.96 ± 0.16	0.075 ± 0.026	0.051 ± 0.006	2.16 ± 0.22	0.395 ± 0.036	0.005 ± 0.004
11	18.7	3.03 ± 0.31	0.183 ± 0.056	0.104 ± 0.017	3.61 ± 0.23	0.409 ± 0.049	0.090 ± 0.011
12	20.0	4.56 ± 0.41	0.423 ± 0.065	0.178 ± 0.029	6.05 ± 0.50	0.584 ± 0.104	0.305 ± 0.047
6 MV photons	0.8	1.00 ± 0.00	0.098 ± 0.008	0.008 ± 0.001	1.00 ± 0.00	0.042 ± 0.005	0.013 ± 0.001

## Discussion

This study presents the microdosimetric characterization of proton RBE in PDAC cell lines, demonstrating that the clinical RBE = 1.1 assumption substantially underestimates proton biological effectiveness in the distal Bragg peak region. For both Panc-1 and COLO 357 FG cells, RBE values exceeded 3 beyond the Bragg peak, reaching 4.6 ± 0.4 (Panc-1) and 6.1 ± 0.5 (COLO 357 FG) at *ȳ_D_* = 20.0 keV/μm. These values are considerably higher than those predicted by current phenomenological RBE models, which typically estimate RBE of 1.3–2.0 in the distal fall-off region (15–17). The difference in RBE between Panc-1 and COLO 357 FG is consistent with prior observations that proton RBE varies across cell lines even within the same tumor type (4), underscoring the need for cell line-specific microdosimetric characterization.

It should be noted that in a clinical spread-out Bragg peak (SOBP) configuration, the superposition of multiple pristine Bragg curves results in a lower *ȳ_D_* distribution throughout the target volume, typically ranging from 5 to 10 keV/μm across the SOBP (18), and the corresponding RBE values would be lower than the peak values reported here. The broad and heterogeneous proton energy spectrum in a clinical SOBP configuration demands the use of detailed microdosimetric spectrum, instead of the mean value of *ȳ_D_*, for accurate microdosimetric characterization of RBE.

The most notable finding is the linear dose-response of COLO 357 FG to protons, despite retaining a clear LQ response to 6 MV photons. This behavior resembles the linear dose-response widely observed for high-LET carbon ion irradiation (12), suggesting that proton-induced damage in COLO 357 FG may be less amenable to repair. The underlying mechanism warrants further investigation, including direct measurement of DNA damage and repair kinetics under matched photon and proton irradiation conditions. Importantly, this finding may have implications for identifying patients with PDAC who are most likely to benefit from proton therapy.

In the current proton therapy practice, the distal Bragg peak region inevitably traverses normal tissues distal to the target, and the assumption of RBE = 1.1 may underestimate the biological dose to these structures. However, treatment plan optimization strategies that intentionally increase the proportion of low-energy, high-lineal energy protons in the Bragg peak region within the tumor volume represent a promising next step toward translating these findings into clinical benefit. Microdosimetry-guided treatment planning for PDAC deserves investigation as a strategy to improve the therapeutic ratio.

Limitations of this study include the use of two cell lines and a crystal violet proliferation assay rather than clonogenic survival. Accordingly, the RBE values reported here should be interpreted as crystal violet-derived, proliferation-based RBE estimates rather than conventional clonogenic RBE values, as crystal violet does not distinguish permanent reproductive inactivation from prolonged growth arrest or senescence. Experiments with additional PDAC lines and clonogenic endpoints are ongoing and will provide orthogonal validation of the *ȳ_D_*-dependent RBE trends reported here. The high-throughput irradiation platform enables efficient expansion to larger cell line panels. Future work will incorporate machine learning-based GSM^2^ modeling to translate the measured RBE dataset into a predictive microdosimetric framework for PDAC, and *in vivo* validation in PDAC tumor models will be pursued to assess the translational relevance of the observed microdosimetric RBE enhancement.

Recent advances in PDAC systemic therapy, including RAS(ON) inhibitors showing promising survival benefits in clinical trials (19), underscore the importance of complementary local tumor control strategies, and combinatorial approaches integrating high-*ȳ_D_* proton therapy with emerging technologies such as pulsed electric field electroporation (20, 21) are being actively investigated by our group to further enhance treatment efficacy in radioresistant PDAC.

## Conclusions

This study showed that proton biological effectiveness in PDAC cell lines increased substantially beyond the Bragg peak and far exceeds the clinical RBE = 1.1 assumption. The distinct dose-response behavior observed between Panc-1 and COLO 357 FG cells highlights the cell line-dependent nature of proton RBE in PDAC and underscores the inadequacy of a uniform RBE for treatment planning in this radioresistant disease. These findings provide a quantitative microdosimetric foundation for improving proton therapy in PDAC and motivate further investigation into microdosimetry-guided treatment planning and *in vivo* validation.

## Data Availability

The original contributions presented in the study are included in the article/supplementary material. Further inquiries can be directed to the corresponding authors.
